# Wide Distribution of a High-Virulence *Borrelia burgdorferi* Clone in Europe and North America

**DOI:** 10.3201/eid1407.070880

**Published:** 2008-07

**Authors:** Wei-Gang Qiu, John F. Bruno, William D. McCaig, Yun Xu, Ian Livey, Martin E. Schriefer, Benjamin J. Luft

**Affiliations:** * Hunter College of the City University of New York, New York, New York, USA; † Stony Brook University, Stony Brook, New York, USA; ‡ Baxter Innovations GmBH, Orth/Donau, Austria; § Centers for Disease Control and Prevention, Fort Collins, Colorado, USA

**Keywords:** Multilocus sequence analysis (MLSA), *ospC*, Lyme disease, phylogeny, recombination, clonal complexes, research

## Abstract

The A and B clones of *Borrelia burgdorferi* sensu stricto, distinguished by outer surface protein C (*ospC*) gene sequences, are commonly associated with disseminated Lyme disease. To resolve phylogenetic relationships among isolates, we sequenced 68 isolates from Europe and North America at 1 chromosomal locus (16S–23S ribosomal RNA spacer) and 3 plasmid loci (*ospC*, *dbpA*, and BBD14). The *ospC*-A clone appeared to be highly prevalent on both continents, and isolates of this clone were uniform in DNA sequences, which suggests a recent trans-oceanic migration. The genetic homogeneity of *ospC*-A isolates was confirmed by sequences at 6 additional chromosomal housekeeping loci (*gap, alr, glpA, xylB, ackA,* and *tgt*). In contrast, the *ospC*-B group consists of genotypes distinct to each continent, indicating geographic isolation. We conclude that the *ospC*-A clone has dispersed rapidly and widely in the recent past. The spread of the *ospC-*A clone may have contributed, and likely continues to contribute, to the rise of Lyme disease incidence.

## Abstract 2

We found substantial population differentiation and recent trans-Atlantic dispersal of a high-virulence *B. burgdorferi* clone.

Multilocus sequence typing (MLST) is the use of DNA sequences at multiple housekeeping loci to characterize genetic variations of natural populations of a bacterial pathogen (*1*,*2*). MLST studies showed that local populations of a bacterial species typically consist of discrete clusters of multilocus sequence types called “clonal complexes,” rather than a multitude of randomly assorted genotypes (*2*). Remaining to be tested are how such factors as natural selection, low recombination rate, and genetic drift due to geographic structuring contribute to the formation and maintenance of these clonal complexes in natural bacterial populations (*3*,*4*). Recently, a multilocus sequence analysis approach was proposed to reconstruct phylogenetic histories of bacterial clonal complexes by using concatenated sequences of housekeeping genes when within-loci and between-loci recombinations are infrequent (*5*).

Lyme disease is a multisystem infection, with inflammatory complications that commonly affect the skin, joints, and central nervous system in humans (*6*). Its causative agent, *Borrelia burgdorferi*, a spirochete that parasitizes vertebrates, is transmitted by hard-bodied ticks throughout the temperate zones of the Northern Hemisphere (*7*). Although humans are accidental hosts of *B. burgdorferi,* Lyme disease is the most common vector-borne disease in the United States with >20,000 annual reported cases, 93% of which occurred in 10 northeastern, mid-Atlantic, and north-central states (*8*). Small mammals such as white-footed mice (*Peromyscus leucopus*) and eastern chipmunks (*Tamias striatus*) serve as the main reservoirs of *B. burgdorferi* (*9*,*10*). In Europe, *B. burgdorferi* is transmitted by *Ixodes ricinus* ticks (*11*) and is carried by a large variety of hosts, including birds and small- to medium-sized mammals (*12*).

*B. burgdorferi* sensu stricto is the primary pathogen of Lyme disease in the United States and is the only pathogenic genospecies that causes Lyme disease in both North America and Europe. More than 12 distinct outer surface protein C (*ospC*) major sequence types coexist in local *B. burgdorferi* sensu stricto populations in the northeastern United States (*13*–*15*). Sequence variability at *ospC* is the highest among known genomic loci and is strongly linked to variations at other genome-wide loci, with occasional recombinant genotypes caused by plasmid exchanges (*16*–*19*).

*B. burgdorferi* sensu stricto intraspecific clonal complexes may differ in their host specificity and degree of human pathogenecity. Different clonal complexes may prefer different host species (*9*). A restriction fragment length polymorphism type of intergenic spacer (IGS) sequence (corresponding to the *ospC-*A and -B groups) is associated with hematogenous dissemination in patients with early stage Lyme disease (*20*,*21*). Four *ospC* clonal complexes (A, B, I, and K groups) were found to be more likely than others to cause disseminated Lyme disease (*22*). Also, an association of *ospC* clonal types with invasive disease in humans has been found in other pathogenic genospecies such as *B. afzelii* and *B. garinii* (*23*,*24*). However, additional *ospC* clonal types have been isolated in patients with invasive disease (*14*).

Previous molecular assays found a close relationship and overlapping genotypes between the European and North American populations (*25*–*27*). These authors found greater genetic diversity among American strains than European strains and proposed a North American origin for this genospecies. Although these studies provided the first evidence for recent intercontinental migrations, they left the phylogenetic relationships among clonal complexes unresolved because of the use of either anonymous genome-wide markers (e.g., arbitrarily primed PCR), genes with a high recombination rate (e.g., *ospC*), or sequences at a single locus. A phylogeographic approach with multiple molecular markers provides a more robust inference on population history (*28*). Here we obtained a well-resolved phylogeny of *B. burgdorferi* sensu stricto clonal complexes by using multilocus sequence typing at housekeeping loci as well as loci under adaptive evolution. We found evidence of genetic endemism, recent migration events, and recombinant genomic types. In fact, the highly pathogenic *ospC-*A clone seems to have spread rapidly in recent years to infect a broad range of host species in 2 continents.

## Materials and Methods

### *B. burgdorferi* Isolates and DNA Isolation

The *B. burgdorferi* sensu stricto isolates were obtained from clinical and tick specimens and cultures from animals in the United States and Europe and maintained as frozen stocks at –70°C (Table 1). For in vitro propagation, a small amount of frozen culture was scraped from the surface of each sample with a sterile inoculating loop and injected into complete Barbour-Stoenner-Kelly II medium (Sigma-Aldrich Corp., St. Louis, MO, USA). Spirochetes were then cultivated at 34°C. All cultures used in this study had undergone a maximum of 2 in vitro passages after recovery from frozen stock. For isolation of genomic DNA, 10 mL of low-passage log-phase bacteria was harvested by centrifugation at 10,000 rpm for 30 min at 4°C. The bacterial pellet was washed twice with Tris-Cl buffer (10 mmol/L Tris [pH 7.5], 100 mmol/L NaCl), and resuspended in 430 μL TES (10 mmol/L Tris [pH 7.5], 100 mmol/L NaCl, 10 mmol/L EDTA). Subsequently, 10 μL of freshly prepared lysozyme (50 mg/mL), 50 μL Sarkosyl (10%), and 10 μL proteinase K (10 mg/mL) were then added, and the mixture was incubated at 50°C overnight before RNase treatment. After incubation, DNA was extracted with phenol/chloroform and chloroform, precipitated with ethanol, and finally resuspended in TE buffer (1 mmol/L Tris [pH7.5], 1 mmol/L EDTA).

**Table 1:** *Borrelia burgdorferi* isolates*

Isolates studied†	*ospC* type‡	Biologic origin	US frequency§	EU frequency
B31, CS1, CS2, CS3, 132a, 132b, IP1, IP2, IP3, Ho, HB1, Lenz, L65, PKa2, HII	A	*Ixodes scapularis*, human	6 (New York)	13 (France, Austria, Germany, Italy, Russia)
N40, 88a, 167bjm, SD91, NP14	E	*I. scapularis*, human	3 (New York)	6 (Hungary)
136b, 163b, 297, CS6, CS9, OEA11	K	*I. scapularis,* human	6 (New England)	1 (Hungary)
109a, 160b, 64b, CS7, MI415¶	B1	*I. scapularis,* human, *Peromyscus leucopus*	5 (New York, Michigan)	0
JD1	C	*I. scapularis*	1 (Massachusetts)	0
121a	D	Human	1 (New York)	0
MI407	F	*P. leucopus*	1 (Michigan)	0
72a	G	Human	1 (New York)	0
156a, 156b, MI403, MI411	H	Human, *Tamias striatus*	4 (New York, Michigan)	0
86b, 97b, MI409¶	I	Human, *T. striatus*	3 (New York, Michigan)	0
118a	J	Human	1 (New York)	0
CS8, 80a, MI418¶	N	*I. scapularis,* human, *P. leucopus*	3 (New York, Michigan)	0
94a, CS5	U	Human, *I. scapularis*	2 (New York)	0
Bol12, VS219,¶ Lx36, ZS7	B2	*I. ricinus*, human	0	17 (Finland, Denmark, Switzerland, Italy, Austria, Slovakia, Germany)
Y1, Y10, 217–5, Bol6, Z6	L	*I. ricinus*	0	10 (Finland, Poland, Italy, Austria)
Fr-93/1, Bol15, Bol25, Bol27	Q	*I. ricinus*, *human*	0	4 (Poland, Italy)
Bol26,¶ Z9, PO7	S	*I. ricinus,* human	0	3 (Italy, Austria)
Bol29, Bol30	V	Human	0	15 (Italy, Switzerland, Slovenia, Germany)
SV1	X	*I. ricinus*	0	1 (Finland)
Ri5	W	*I. ricinus*	0	1 (Finland)

*ospC, outer surface protein C; US, United States; EU, European Union. †Isolates subjected to multilocus sequence typing analysis. ‡Type names follow (*13*), except that B was split to B1 and B2, and 3 new types (V, X, W) were assigned to European isolates. §Number and geographic origins of an *ospC* type in our collection. ¶Isolates showing evidence for plasmid-chromosome recombination.

### Genomic Markers, PCR Amplifications, and DNA Sequencing

PCR amplifications were attempted at 4 genomic loci for all isolates and at 6 chromosomal housekeeping loci for a genetically representative subset of isolates (Table 2). The IGS locus was chosen for its phylogenetically informative polymorphisms (*16*,*20*). The IGS locus and 6 housekeeping genes (*gap, alr, glpA, xylB, ackA,* *tgt*) were approximately evenly distributed on the main chromosome based on the B31 genome (*29*). The 3 plasmidborne loci were selected for their high sequence variability and for the absence of close paralogs based on a genome comparison (*17*,*19*). IGS sequences were amplified by using a nested PCR procedure (*30*). Because of high sequence variability, *dbpA* sequences were amplified by using 2 alternative forward primers. PCR amplification was performed in 50 μL containing 200 mmol/L of each dNTP, 2.0 mmol/L MgSO_4_, 2.5 U of Platinum Taq DNA polymerase High Fidelity (Invitrogen, Carlsbad, CA, USA), 0.5 μmol/L of each primer, and 100 ng of genomic DNA template. Following denaturation at 94°C for 1 min, samples underwent 30 cycles of denaturation at 94°C for 30 s, annealing at 55°C for 30s, initial extension at 68°C for 1.5 min, and a final extension step at 68°C for 10 min. PCR products were purified by GFX chromatography (Amersham Pharmacia Biotech, Inc., Piscataway, NJ, USA), resolved by agarose gel electrophoresis, and visualized by ethidium bromide staining. Purified amplicons were sequenced by using standard dideoxy terminator chemistry as outlined below with the forward and reverse PCR primers. Absence of specific PCR products, indicating potential absence of particular genetic loci or plasmids, was confirmed by follow-up amplifications of the flanking DNA segments.

**Table 2:** Genomic markers and PCR primers

Locus*	Primer sequence (5′ → 3′)†	Location‡
BB0057 (*gap*)	F-ATGAAATTGGCTATTAATGG, R-TTGAGCAAGATCAACCACTC	Main chromosome (52.5 K)
BB0160 (*alr*)	F-ATGTATAATAATAAAACAATGG, R-ATTTTCTCTTTTCGTATTTTCC	Main chromosome (160 K)
BB0243 (*glpA*)	F-ATGGAGGAATATTTAAATTTC, R-GTTCATTTTTCCACTCTTC	Main chromosome (249 K)
IGS (*rrs-rrlA*)	1st round§: F-GGTATGTTTAGTGAGGG, R-GGTTAGAGCGCAGGTCTG; 2nd round: F-CGTACTGGAAAGTGCGGCTG, R-GATGTTCAACTCATCCTGGTCCC	Main chromosome (444 K)
BB0545 (*xylB*)	F-ATGAATGCTCTTAGTATTG, R-CCCGTTAACAAATAGAC	Main chromosome (555 K)
BB0622 (*ackA*)	F-TTGTCAAATACAAAAGG, R-AATGTCTTCAAGAATGG	Main chromosome (649 K)
BB0809 (*tgt*)	F-ATGTTTAGTGTAATCAAGAATG, R-ATCGAAATTTTCCTCTTCATAC	Main chromosome (855 K)
BBA24 (*dpbA*)	F1-TAATGTTATGATTAAATG, F2-ATGAATAAATATCAAAAAAC, R-GAAATTCCAAATAACATC	lp54
BBB19 (*ospC*)	F-CCGTTAGTCCAATGGCTCCAG, R-ATGCAAATTAAAGTTAATATC	cp26
BBD14	F-ATGATAATAAAAATAAAAAATAATG, R-ATTTTGATTAATTTTAATTTTGCTG	lp17

*B31 open reading frame (gene) names. IGS, intergenic spacer. †F, forward; R, reverse. ‡Approximate starting positions on the B31 genome (*29*). §Source (*30*).

Automated DNA sequencing of both strands of each fragment was performed by the Stony Brook University Core DNA Sequencing Facility (Stony Brook, NY, USA) by using the dye-terminator method with the same oligonucleotide primers used for PCR amplification or, where required, appropriate internal primers. Sequences were inspected and assembled with the aid of the Sequencher program (Gene Codes, Inc., Ann Arbor, MI, USA). DNA sequences were analyzed by using the BLASTN program through GenBank at the National Center for Biotechnology Information (www.ncbi.nlm.nih.gov). Nucleotide and protein sequence alignments were performed with MacVector version 6.5 (MacVector, Inc., Cary, NC, USA). New sequences were deposited to GenBank under accession nos. EF537321–EF537573.

### Phylogenetic Inference and Tests of Population Differentiation

The IGS sequences were used to resolve intraspecific phylogenetic relationships among *B. burgdorferi* isolates (*16*,*20*). Two highly divergent tick isolates from Finland (SV1 and Ri5) were used as outgroups for rooting the phylogenetic tree. IGS sequences were aligned by using ClustalW (*31*). A Bayesian majority-rule consensus tree was estimated by using MrBayes (version 2.1) (*32*) as described previously (*19*). Sequences at the 3 plasmid-borne protein–coding loci were translated into protein sequences and aligned in a pairwise fashion with ClustalW (*31*). Nucleotide alignments were obtained according to the protein alignments. Neighbor-joining trees based on pairwise nucleotide sequence distances were inferred by using PHYLIP (*33*) and plotted by using the APE package of the R statistical package (*34*). Genetic differentiation among geographic populations was tested by using the analysis of molecular variance (AMOVA) method implemented in the software package Arlequin 3.1 (*35*). The 6 housekeeping genes were used to infer the overall within- and between-genospecies phylogeny. Sequences of strains B31 and PBi (*B. garinii*) were downloaded from GenBank (*29*,*36*). Sequences of N40, JD1, DN127 (*B. bissettii*), and PKo (*B. afzelii*) were from draft genomes (S. Casjens, pers. comm.). The 6 alignments were concatenated and tested for the presence of gene conversion by using GENECONV with the “within-group fragments only” option (*37*). Two approaches, a Bayesian method with codon site-specific evolutionary rates (using MrBayes) and the other maximum likelihood method with 100 bootstrapped alignments (using DNAML in PHYLIP) (*33*), were used for phylogenetic reconstruction based on concatenated sequences. Branch supports were measured by the posterior probabilities in the Bayesian method and the bootstrap values in the maximum likelihood method.

## Results and Discussion

### AMOVA Tests of Geographic Differentiation

We sequenced 68 isolates (including 30 from northeastern United States, 6 from the midwestern United States, and 32 from Europe) at a single chromosomal locus (IGS) and 3 plasmid loci (*ospC*, *dbpA*, and BBD14). Using AMOVA, we evaluated the genetic differentiation among geographic samples and found significant genetic differentiation between the North American and European populations at IGS, *ospC*, and *dbpA*, but not BBD14 (Table 3). Among these loci, IGS is the most informative in reflecting the effect of genetic drift caused by geographic isolation because sequence variations at IGS are likely to be selectively neutral. In addition, IGS is on the main chromosome and less likely to be subject to gene conversion. Genetic variations at 3 plasmid loci are more likely to be influenced by natural selection such as adaptation to local vector and host species. Also, plasmid genes are more likely to be transferred so that footprints of geographic isolation might be obscured by gene flow between populations. Natural selection can both enhance and reduce geographic differentiation. With adaptation to local habitats, natural selection acts to enhance the geographic divergence, especially at target loci. On the other hand, diversifying selection within populations inflates within-population diversity, which results in lack of differentiation within populations relative to the within-population polymorphism.

**Table 3:** Analysis of molecular variance results*†

Locus	Molecular variance, %			Nucleotide diversity, π		Fixation index (*F_ST_*)‡
	Between continents	Within continents		North America	Europe	
IGS	19.5	80.5		0.0253	0.0243	0.1952§
*ospC*	3.13	96.87		0.2066	0.1900	0.0313¶
*dbpA*	26.5	73.5		0.1480	0.0999	0.2650§
BBD14	2.54	97.46		0.0834	0.1333	0.0254 (NS)

*IGS, intergenic spacer; *ospC*, outer surface protein C; NS, not significant (p>0.05). †Results were obtained by using Arlequin 3.1 (*35*). Samples were 66 IGS sequences divided into 2 continental populations: North America (36 sequences from New York, Connecticut, Massachusetts, and Michigan) and Europe (30 sequences from Italy, Austria, France, Germany, Switzerland, Poland, Hungary, Slovenia, and Finland). Two outgroup sequences (SV1 and Ri5) were excluded from the European sample. Genetic distances between haplotypes were based on the Kimura 2-parameter model. ‡Levels of significance were obtained by 1,000 permutations. §p<0.001. ¶0.01<p<0.05.

**Figure 1:**
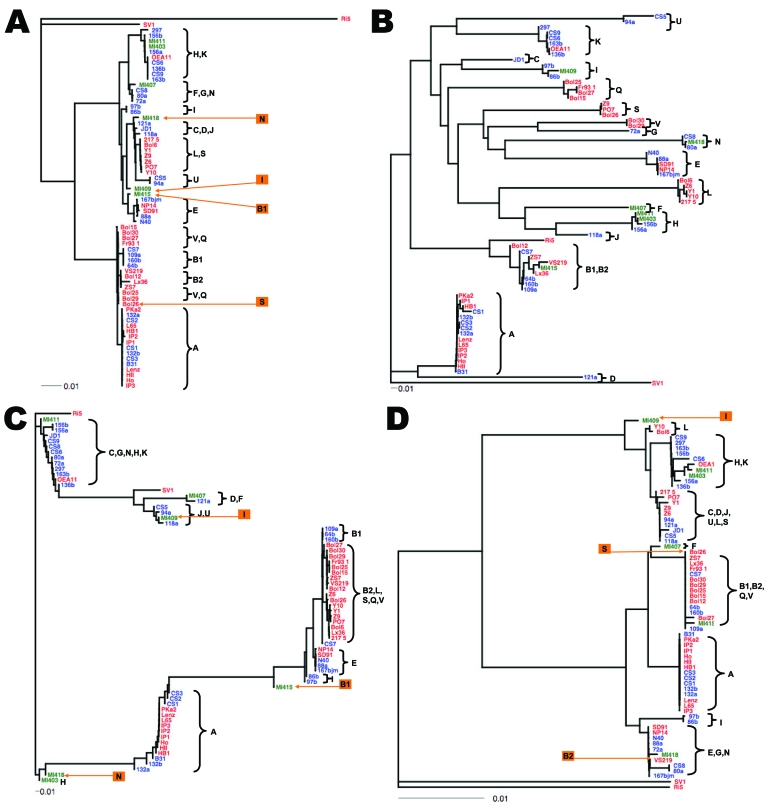
Gene trees showing nucleotide sequence clusters of 68 *Borrelia burgdorferi* isolates at 1 chromosomal locus (panel A: *rrs-rrlA* spacer, or intergenic spacer [IGS]) and 3 plasmid loci (panels B, C, and D: *ospC* on cp26, *dbpA* on lp54, and BBD14 on lp17, respectively). Trees were inferred based on nucleotide sequence alignments and were rooted by using the Ri5, SV1, or both, sequences as outgroups. The DNADIST and neighbor-joining programs of the PHYLIP package (*33*) were used for distance calculation and the APE software package (*34*) was used for tree plotting. Isolates were grouped as clonal groups (A through U), which are named by their typical *ospC* alleles. Five isolates (Bol26, VS219, MI409, MI415, and MI418) showing atypical allelic associations with *ospC* alleles, likely caused by recombination, were labeled in orange. Red, European isolates; blue, northeastern US isolates; green, midwestern US isolates. Scale bars indicate number of nucleotide substitutions per site.

The low level of geographic differentiation at *ospC* showed the divergence-reducing effect of natural selection. Genetic variability of *ospC* is as high within populations as between populations and is caused by diversifying natural selection (*9*,*13*). In such a case, summary statistics such as AMOVA fixation index (*F_ST_*) are misleading because sequence cluster analysis showed that most *ospC* alleles have geographically restricted distributions (Figure 1, panel B). The insignificant AMOVA result at BBD14 might be due to a similar effect of high within-population polymorphisms as a result of diversifying selection. In contrast, *dbpA* showed the divergence-enhancing effect of natural selection. The *dbpA* locus showed the highest level of geographic differentiation, owing to a shared allelic type among B2, L, S, Q, and V clonal groups in Europe (Table 3; Figure 1, panel C). An adaptive sweep likely has homogenized these divergent European lineages at *dbpA*.

In summary, on the basis of the neutral genetic variations at IGS, we conclude that the European and North American populations of *B. burgdorferi* sensu stricto have diverged significantly because of genetic drift. Plasmid genes evolved independently and showed various effects of adaptive divergence and diversifying selection. At all 4 loci, genetic variations within the 2 continents contributed to most (>70%) of the total sequence diversity, which suggests recent common ancestry, migration, or both, between the European and North American populations.

### Endemic and Shared *ospC* Alleles

Gene trees showed more detailed pictures of geographic variations at each locus (Figure 1). Among the 17 major sequence groups of *ospC,* 2 minor sequence variants of major-group allele B were geographically distinct and thereby named B1 in North America and B2 in Europe. Three *ospC* alleles (A, E, and K) were observed in both continents, 5 (B2, S, L, Q, and V) exclusively in Europe (not including the outgroup Ri5 and SV1 alleles), and 10 (B1, C, D, F, G, H, I, J, N, and U) exclusively in North America (Table 1). Although the sample sizes of the North America isolates were small, the same set of *ospC* alleles has repeatedly been identified in surveys of natural populations (*14*–*16*,*38*). These isolates are therefore a reasonably complete representation of *ospC* diversity in North America. How well our European samples represent the overall *ospC* diversity in Europe is less certain because the European isolates were from an archived collection rather than from systematic surveys of natural populations. For instance, *ospC* alleles J, P, and R have been identified in Europe (*26*). Nonetheless, *ospC-*A appeared to be the only allele that is highly prevalent on both continents (Table 1). An earlier study showed that *ospC-*A and *ospC*-B alleles existed in both continents, whereas other *ospC* alleles were geographically distinct (K, J, F in North America and P, Q, R, S in Europe) (*24*). Our results further suggested that the *ospC-*B clonal group had 2 geographically distinct subtypes (Figure 1, panel B).

### Recombinant Genotypes

Previous MLST studies showed complete linkage between *ospC* and other loci on plasmids or the main chromosome in the North American populations (*15*,*16*). This finding is consistent with our study, in which allelic types at IGS, *dbpA*, and BBD14 of the 68 isolates were almost entirely predictable from their *ospC* types. Because of the nearly complete linkage between *ospC* and a locus, individual clonal complexes could conveniently be named after their *ospC* alleles. However, 5 isolates showed alleles at non-*ospC* loci inconsistent with allelic types typically associated with their *ospC* alleles, including MI409, MI415, and MI418 from the midwestern United States and Bol26 and VS219 from Europe (Figure 1). Because these genotypes were new combinations of allelic types found elsewhere, they are more likely to be recombinant genotypes caused by plasmid exchanges, rather than locally evolved new genotypes (*17*). Notably, these probable recombinants were from samples from either the midwestern United States or Europe, and none were from the intensively surveyed northeastern United States. A higher number of clones in the northeastern United States than elsewhere could be understood because *B. burgdorferi* populations in that region are evolutionarily young and show an epidemic population structure (*15*,*19*). On the basis of the presence of allele types at 4 loci, we determined preliminarily that Bol26 is a group Q or V clone with a transferred *ospC-*S allele because Bol26 clustered with group Q and V isolates at IGS, *dbpA*, and BBD14 (Figure 1). By the same reasoning, VS219 is a group B2 clone with a transferred BBD14 allele. We are currently investigating the donor and recipient genomic types of these recombinant isolates by sequencing 6 additional loci.

### Recent Trans-Oceanic Dispersals

**Figure 2:**
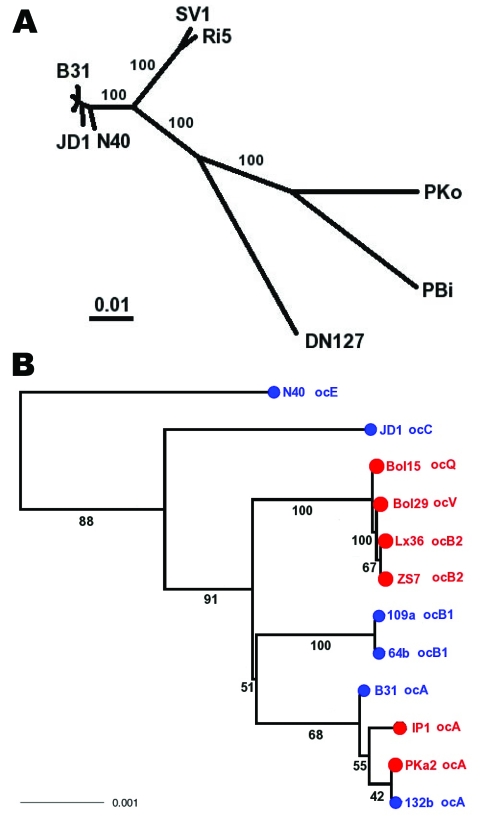
Species phylogeny based on concatenated sequences at housekeeping loci. Seventeen isolates include 1 *Borrelia garinii* strain (PBi), 1 *B. afzelii* strain (PKo), 1 *B. bissettii* strain (DN127), 2 strains of an unnamed genomic species (SV1 and Ri5), and 12 *B. burgdorferi* sensu stricto isolates. These strains were selected for reconstructing interspecies phylogeny (hence species samples), as well as for resolving the clade containing clonal groups A and B (A, B1, and B2 are represented by 2 isolates). Sequences at 6 chromosomal housekeeping loci (*gap, alr, glpA, xylB, ackA,* and *tgt*) were obtained for each strain, with B31 and PBi sequences from published genomes (*29*,*36*), N40, JD1, PKo, and DN127 sequences from draft genomes (S. Casjens et al. pers. comm.). Sequences of the remaining strains were obtained by direct sequencing. The total length of concatenated alignment is 7,509 nt. A) Consensus of maximum likelihood trees obtained by using DNAML of the PHYLIP package (*33*). Branch support values are based on 100 bootstrapped replicates of the original alignment. B) Enlarged view of *B. burgdorferi s*ensu stricto subtree. Tips were colored by geographic origin of the isolate (blue, North America; red, Europe) and were labeled with *ospC* major-group allele type. Scale bars indicate number of nucleotide substitutions per site.

Three clonal complexes (A, E, K) are distributed in both continents (Table 1). For the A clonal group, 6 isolates from the United States and 11 isolates from Europe were sequenced at 4 loci. The 4-locus sequences of the isolates between the 2 continents were identical (Figure 1). Thus, the A clonal complex likely was dispersed across the Atlantic Ocean rather recently. To verify the genetic homogeneity of group A isolates from the 2 continents, we randomly selected 4 group A isolates (B31 and 132b from the United States; IP1 and PKa2 from Europe) for further sequencing at an additional 6 chromosomal loci. No fixed sequence differences between 2 continental samples were found, which lends further support for the recent trans-oceanic migration of the A clone (Figure 2). Similarly, the 4-loci sequences of E and K isolates between the 2 continental samples were identical, indicating recent migration of these clonal groups as well (Figure 1). However, the E and K groups seemed less prevalent in Europe than the A group (Table 1). Because individual ticks and hosts are commonly infected with multiple *B. burgdorferi* clones, any migration, whether by natural or human-facilitated mechanisms, is likely to involve a mixture of clonal groups, rather than a single clone. Upon their arrival, however, clonal groups may differ in their ability to colonize a new niche consisting of novel vector and host species. By this reasoning, the A clone is the most ecologically successful strain, able to thrive in a new niche with little genetic change. This conclusion is supported by surveys that showed a broad range of host species for this clonal group (*9*,*10*).

We could not determine conclusively the direction, timing, or number of the trans-oceanic dispersals. Assuming that the chromosomal gene tree in Figure 2, panel B, is an accurate representation of the phylogeny of these clonal groups, a parsimonious scenario is that an early migrant from Europe was the ancestor of the North American clade consisting of the A and B1 groups, and a more recent migration has introduced the A group to Europe. However, none of the basal branches of this gene tree was well supported (Figure 2). Multilocus sequencing of more loci, especially rapidly evolving plasmid loci, of group A isolates will help find more conclusive answers to these questions. To estimate the time of the A clone migration, we noted that no fixed differences in nucleotides occurred within a total of 11,167 aligned bases at 7 chromosomal and 3 plasmid loci. If one assumes a neutral evolutionary rate on the order of 1 substitution per site per million years, the Poisson zero-term probabilities that no fixed difference has occurred within 11,167 bases in the past 50, 100, and 200 years are 0.33, 0.10, and 0.011, respectively. Therefore, the trans-oceanic migration of clone A likely occurred more recently than 200 years ago. More realistic estimates would depend on studies of the neutral mutation rate and generation time of *B. burgdorferi* in the wild.

### Phylogenetic Heterogeneity of Group B Isolates

The *ospC-*B clonal group is another highly virulent strain identified by association studies (*20*–*22*,*24*). Initially, group B seemed to be another clone that is distributed in both continents with a few sequence differences at IGS and *ospC* (Figure 1). Sequencing at additional 6 housekeeping loci, however, showed deep phylogenetic heterogeneity of the B group, while the A group remained homogeneous (Figure 2). The 2 B clonal complexes (B1 in North America and B2 in Europe) do not form a monophyletic clade (Figure 2). Rather, B2 clusters with other European clones (V and Q). Also, clones B1 and A, the 2 closest North American relatives, do not form a well-supported clade (only 51% bootstrap support). Clearly, unlike the A clone, the bicontinental distribution of the B clone is not due to recent migration. Sharing of similar *ospC* B alleles between the 2 continents may be due to stabilizing selection or lateral transfer. Because few synonymous changes have occurred between the B1 and B2 alleles, lateral transfer is a more likely cause.

The B2/Q/V showed as a European clade with nearly uniform chromosomal sequences, although it had highly divergent *ospC* alleles (Figure 2). This evidence, based on chromosome-wide genes, strengthens the conclusions of an earlier study that adaptive, large sequence variations at *ospC* are associated with incipient genome divergence (*19*).

Finally, the overall genospecies phylogeny based on MLST showed that the 2 European isolates (Ri5 and SV1) that we used as outgroups may be a new genospecies (Figure 2). This phylogeny is robust because tests of recombination using GENECONV showed no statistically significant gene conversion within the 6 chromosomal housekeeping loci (*37*). The hypothetical genospecies represented by Ri5 and SV1 is more closely related to *B. burgdorferi* sensu stricto than *B. bissettii* (represented here by DN127) is to *B. burgdorferi* sensu stricto. Thus, the MLST phylogeny suggests a possibility that Europe, rather than North America, may be the origin of *B. burgdorferi* sensu stricto, despite a higher contemporary genetic heterogeneity in North America than in Europe.

## Conclusions

To summarize, the present study used 7 chromosomal loci (IGS and 6 housekeeping genes) to reconstruct the intra- and interspecific phylogeographic histories of *B. burgdorferi* sensu stricto. Although the standard MLST scheme based on housekeeping genes enables estimates of recombination and mutation rates as well as intraspecific phylogenies (*2*,*5*), our approach of including plasmidborne loci under positive selection helped identify the selective causes of bacterial lineage divergence. Our results showed significant endemic lineage diversification among regional populations, discovered recombinant genotypes, and strongly indicated migrations between North American and European populations in modern times. The highly pathogenic clonal complex A has a prominent presence in both continents, which suggests its success in finding ecologic niches that enable it to infect a broad range of host and vector species. The same genetic basis of the ecologic invasiveness of the *ospC*-A clone may be underlying its high virulence to humans. The emergence of Lyme disease in North America since the 1970s has been attributed to an increasing overlap of human and *B. burgdorferi* habitats (*39*). On the basis of our evidence of migration events, we propose that the trans-oceanic dispersal and colonization of ecologically highly successful clonal complexes (e.g., the A group) may also have played a substantial role.
